# Bad Teeth and Their Effect on the Laboring Man’s Efficiency*Read before the Section on Preventive Medicine and Public Health at the Sixty-Seventh Annual Session of the American Medical Association, Detroit, -June, 1916. Reprinted from the *Journal of A. M. A.*, January 13, 1917.

**Published:** 1917-02

**Authors:** Carl E. Smith

**Affiliations:** Akron, Ohio


### BAD TEETH AND THEIR EFFECT ON THE LABORING MAN'S EFFICIENCY.*

BY CARL E. SMITH, D.D.S., AKRON, OHIO.

Efficiency from a health standpoint is difficult to determine. We know, for instance, that if the water supply of a community is pure, the community is saved economic loss from typhoid; the same can be said of the milk supply and of all the precautions necessary to keep a community in health.

This is true from a dental standpoint. If we teach a large body of people mouth hygiene we can not say how much better their health will be, or how much we have saved them through the prevention of mouth and systemic diseases.

In the past sixteen months I have made thirty thousand mouth examinations, for seventeen thousand Americans and thirteen thousand foreigners. Of this number, ninety-six per cent. are in need of dental service; only four per cent., have clean, healthy mouths; nine per cent. are without cavities and could be made healthy by a thorough cleaning; the balance have all the pathologic conditions known to dentistry, pyorrhea pockets, decayed and abscessed teeth, mucous plaques with all the virulent bacteria ready to cause disease the moment there is a lowering of the resistance. The danger from a dirty mouth is quite generally recognized, practically all contagious diseases being transmitted by the nose or mouth and the mouth is the more dangerous of the two. In a mouth free from caries, but with deposits of calculi and mucous plaques, gingivitis develops, and because the gums are

---

* Read before the Section on Preventive Medicine and Public Health at the Sixty-Seventh Annual Session of the American Medical Association, Detroit, June, 1916. Reprinted from the *Journal of A. M. A.*, January 13, 1917.

painful and bleed the man gives up mastication, and food passes to the stomach carrying pathogenic organisms. The stomach can not do double duty, and in time disease results. And so by teaching oral hygiene to the laboring man and he in turn carrying the idea to his family, we are cutting down disease, not only in the factory, but in the community.

Suppose an epidemic of scarlet fever breaks out in a city. The health authorities find these cases along a certain milk route, and they look up the farms on which this milk is produced and find a case of scarlet fever. I have never heard a satisfactory explanation of how that first case started, apparently spontaneously. Could it not be a case of lowered vital resistance from some cause and a dirty mouth, in which the germs of scarlet fever predominate? Whether this be true or not, the teaching of oral hygiene to the laboring man is important if it does nothing more than prevent caries, pyorrhea and other mouth diseases.

Now in thirty thousand mouths we find about sixty thousand cavities, and I intend to show what one cavity means to the average laboring man. First, there is the slight opening of the interdental space; food packs into the space, irritating the gum. Quite naturally he gives up mastication on that side of the mouth; caries progresses until the pulp is exposed, the tooth aches, and he goes home from work, often purchasing a bottle of whisky on the way. I have had cases in which the patients have admitted spending their last cent for whisky and had to borrow money to go to the dentist.

The pulp in the tooth may die in twenty-four hours, or it may stop aching, only to start again in a few days. If the pulp dies, in a period of time we have the acute alveolar abscess and at last in desperation he goes to the dentist and has the tooth extracted. During this period of time, due to disuse, quantities of filth have collected over the other teeth on that side of the mouth, caries has started in the tooth next to the one extracted, and within a few months he again goes through with the same experience. By this time the other teeth have become decalcified, and within a comparatively short time he loses every tooth on that side of the mouth, and it is easy to understand this man could not be marked one hundred per cent. in efficiency during this period. If we admit the teeth to be necessary to mastication and digestion, we know that the employe can not enjoy the good health he would have had, had we been able to prevent that first cavity or at least to persuade him to have dental service before it was too late.

In thirty thousand mouths we find about eighteen thousand extractions necessary, and experience shows these teeth to be practically all abscessed. This is a difficult problem in efficiency, especially so if the findings of the research commission of the National Dental Association in regard to the relation of infections of the mouth to general systemic disorders prove only part truth, but the indications are at the present time that these apparently unimportant foci of infection are the cause of many disorders.

The average dentist knows little at the present time regarding the effect of these foci of infection. If it is arthritis, stomach ulcer, heart lesions, pernicious anemia or any of the nervous diseases of a neuralgic type, the medical man is consulted. If it is true that these foci of infection are causing these obscure diseases, it is time for the dentist to learn more of medicine, so that he may hold intelligent consultation with the physician, and the physician should seek the consultation of intelligent dentists. One case along this line I wish to report. After working for the company for six years this man laid off in November, 1913, to have a mastoid operation. In July, 1915, he applied for re-employment. Physical examination showed ankylosis of the knee and poor physical condition. The mouth examination showed a very bad case of pyorrhea and several abscessed teeth. His mouth may not have been the cause of his trouble, but the surgeon overlooked the possible cause, and the patient should have been sent to a dentist. Had this been done, he might at this time be an efficient workman. On the other hand, there are no doubt a good many of your patients whom you are burying on account of poor dentistry.

I wish to report a case which to my mind shows how bacteria are carried by the blood stream. A man injures a tooth in such a way as to kill the pulp. After a period of time an alveolar abscess develops. I have seen a number of these cases without a cavity in the tooth, no pyorrhea, and gums in a healthy condition. There is only one way that infection could occur. The bacteria were carried there by the blood stream from some focus of infection. If they are carried to the teeth, it follows naturally that they are carried from these blind alveolar abscesses.

If a man presents himself to our physical examiners with a suppurating toe-nail or infected finger nail, he is promptly rejected as being unfit for employment, likewise if he has an acute alveolar abscess with a swollen jaw he is rejected until the cause has been removed and the swelling subsides, but we can not reject these men with eighteen thousand necessary extractions, knowing or at least suspecting the effect on their efficiency; if we did, it would mean the rejection of twenty-five per cent. of all people applying for employment. The best we can do is to advise the removal of these teeth. In thirty thousand mouths we find forty thousand teeth extracted, and that tells its own story, the inability to properly masticate the food, and you medical men know better than I the results of improper digestion which are bound to follow.

One way we can estimate the loss of efficiency due directly to toothache is by watching the piece-worker. Very often a man engaged in piece-work will say: "Doctor, this tooth has ached for a week." The loss of money to these men runs from three to seven dollars for the week. Men suffering for a day report losses of from thirty-five cents to one dollar and a half.

In thirty thousand mouths we find sixty thousand cavities and eighteen thousand extractions necessary, seventy-eight thousand in all. If each one causes an average loss, through waste of time and cost of repair, of two dollars, it means a cost of one hundred and fifty-six thousand dollars. In New York City last year sixty-seven thousand children failed to be promoted to higher grades because of absence, eighty per cent. of which can be laid to defective teeth, and it costs New York one million thirty-seven thousand six hundred and ninety-six dollars to duplicate a year's schooling to these absentees. These same children in a few years will be employed by our industrial concerns and a large percentage will fail of promotion on account of poor health, due to bad mouth conditions.

What can be done by way of prevention? The first thing necessary is the co-operation of the medical man; he is the first to see the pregnant woman; he should instruct her regarding what to eat to supply the necessary mineral salts to build bone and teeth, and send her to the dentist for instruction regarding the care of her mouth. The dentist should instruct her regarding the mouth of her child when it arrives, also regarding the care of that mouth after the teeth begin to erupt. It is possible to take a child at this time and prevent the formation of cavities, and it is most important that this should be done.

We find at the present time a great many dental clinics in the public schools. This is a good work, but the child is six years of age when he starts school and a great deal of damage has already occurred, so the work of the clinics is largely reparative, but still it is not too late to teach the children oral hygiene. They should be taught how to live, which is vastly more important than certain subjects which have been a part of the school curriculum in past years. If this were done, they would leave school and enter our industrial institutions better equipped to earn a livelihood.

During the past two years dental clinics have been established in a number of large manufacturing concerns. These clinics are operated along different lines, but all with the same general idea in view, that of cooperating with the medical departments in bringing about more satisfactory health conditions.

